# Syndrome de Chilaiditi chez un nouveau-né, à propos d'un cas

**DOI:** 10.11604/pamj.2014.19.239.5059

**Published:** 2014-11-03

**Authors:** Sangwa Milindi Cedrick, Kitembo Feruzi Maruis, Kakinga Zabibu Mireille, Mukonda Sompo Nelly, Muhau Pfutila Patience, Mapatano Shem, Cham Lubamba Chamy, Monga Kalenga Josephine

**Affiliations:** 1 Département de Chirurgie, Université de Lubumbashi, Lubumbashi, Congo; 2 Institut de Recherche en Sciences de la Santé, Antenne de Lubumbashi, Lubumbashi, Congo; 3 Département de gynécologie, Université de Lubumbashi; 4 Département de pédiatrie, Université de Lubumbashi

**Keywords:** Chilaiditi, nouveau-né, féminin, Chilaiditi, newborn, female

## Abstract

L'interposition du colon ou du grêle dans l'espace interhepatodiagramatique est une affection rare. Souvent asymptomatique et de découverte fortuite à la radiographie de l'abdomen, elle est plus rencontrée chez l'adulte de sexe masculin. Dans cet article, les auteurs présentent un cas exceptionnel d'un nouveau- né de sexe féminin porteur de cette anomalie.

## Introduction

Le syndrome de chilaiditi est une pathologie caractérisée par l'interposition du colon ou du grêle dans l'espace interhepatodiagramatique. C'est une pathologie dont les signes cliniques ont été décrits pour la première fois en 1865 par Cantini, et c'est seulement en 1910 que Demetrius Chilaiditi rapport 3 cas de patients avec image radiologique d'interposition du colon entre le diaphragme et le foie [1]. C'est signe baptisé de chilaiditi est toujours de découverte fortuite lors d'un examen radiologique du thorax ou de l'abdomen. Le syndrome de chilaiditi désigne les manifestations cliniques: douleurs abdominales, vomissement, anorexie et constipation liées à ce désordre [2]. Dans la plupart de cas les sujets sont asymptomatiques [3]. L'incidence mondiale de cette malposition varie de 0.025 à 0.28%. Le sexe masculin est plus concerné que le sexe féminin. Cette pathologie est plus rencontrée chez les personnes âgées; elle est rare chez les enfants [4–6] et aucun cas à notre connaissance n'a été rapporté chez un nouveau -né en Rd Congo. Dans cet article, nous vous rapportons un rare cas de syndrome de Chilaiditi chez un nouveau-né de sexe féminin.

## Patient et observation

**Figure 1:**
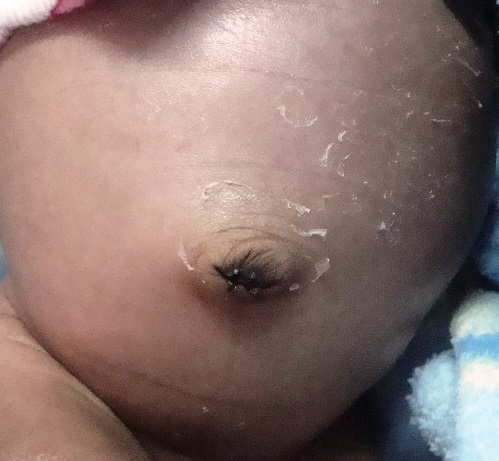
Ballonnement abdominal suite au syndrome de chilaiditi

**Figure 2:**
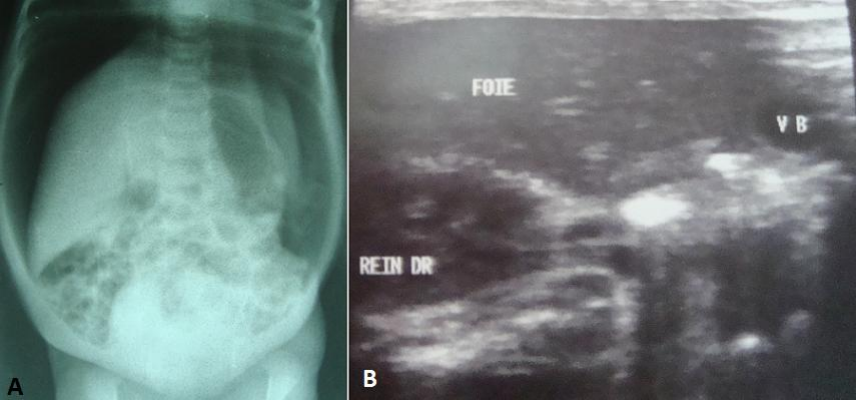
Image radiologique et echographique d'interposition du colon entre le foie et l'hemicoupole diaphragmatique droit (A et B)

Un nouveau-né de 4 jours de sexe féminin a été admis en date du 25 mai 2014 dans le service de néonatologie de l'Hôpital Général Provincial de Référence Janson Sendwe de Lubumbashi, pour pleurs incessants, ballonnent abdominal et vomissement post prandial d'aspect jaunâtre depuis 2 jours. Sa mère, ménagère âgée de 30 ans, P7G6A0D0 signale avoir suivi dès le première trimestre de la grossesse les consultations prénatales. Elle signale avoir développé une infection urinaire au cours du troisième trimestre traitée à l'hôpital. L'accouchement à terme a été eutocique. L'enfant a pesé 2630 g avec un score d'APGAR excellent. Le même jour, le nouveau-né a émis 3 fois le méconium. A notre examen physique, l'enfant n'a présenté aucune malformation apparente. Nous avons noté un ballonnent abdominal (Figure 1), avec un tympanisme periombilicale et une disparition de la matité pré hépatique. L'abdomen était dépressible sans organomegalie avec un péristaltisme conservé. Devant ce tableau clinique, un diagnostic de subocclusion intestinale néonatale a été évoqué et une radiographie de l'abdomen à blanc a été réalisée. Cette derniere a révélé la présence d'une importante hyperclarté sous forme d'un croissant gazeux interhepatodiaphragmatique droit se prolongeant dans la fosse iliaque du même côté, le foie étant refoulé en bas et en dedans vers la région periombilicale (Figure 2). L’échographie réalisée un jour après la radiographie a montré un excès de gaz abdominal semblable à une aerocolie sous diaphragmatique droit en rapport avec le signe de chilaiditi (Figure 2). Les autres organes intraabdominaux étaient intacts. Une canule rectale placée et maintenu pendant 2 jours a permis à l'enfant émettre progressivement les gaz et les selles. Il s'en ai suiviune remissions totale des signes au 10^ème^ jours de naissance. L’équipe médicale a opté pour traitement conservateur.

## Discussion

L'incidence du syndrome de Chilaiditi à travers la population mondiale est estimée entre 0.025 à 0.28%. Cette affection est plus observée chez le sujet adulte de sexe masculin et est extrêmement rare dans l'enfance [1]. Nous n'avons pas rencontré dans la littérature un seul cas rapporté chez le nouveau-né. Notre enfant de 4 jours de sexe féminin constitue un cas exceptionnel.

Du point de vue étiopathogénique, il est établi qu'en situation normale, le développement embryologique du foie ainsi que le moyen de fixation de l'intestin empêche une interposition du colon entre le diaphragme et le foie. Ceci peut cependant survenir au cours de certaines variations anatomiques rarement rencontrées: l'hypotrophie du foie ou l'agénésie du lobe droit du foie, l’élongation du ligament suspenseur du foie; l'allongement du colon (dolichocôlon), la mal fixation ou la mal position du colon, les pathologies congénitales de l'intestin grêle ou du diaphragme avec élévation de l'hemicoupole diaphragmatique droite (éventration), la relaxation ou l'agénésie du ligament suspenseur du mésentère [7–9]. Ces caractéristiques sont remarquées chez 6% des patients à la naissance. Par ailleurs, chez l'adulte d'autres causes sont incriminées dont la cirrhose [10], la constipation chronique, l'augmentation de la pression abdominale (la grossesse) [11], l'obésité [12], l’élargissement des bases de la cage thoracique due à une pneumopathie obstructive chronique avec un large espace dans lequel l'interposition du colon peut survenir [1].

Sur le plan anatomopathologique, c'est le colon transverse en premier suivi de l'angle colique hépatique qui sont les segments du colon les plus rencontrés interposer entre le foie et le diaphragme ou la paroi abdominale, toute fois l'interposition de l'intestin grêle est aussi rapporté[11–13].

Quant à la clinique, l'interposition du colon est souvent un signe radiologique asymptomatique [3, 8, 6] Chez notre nouveau-né, nous avons noté des pleurs incessants, un ballonnement abdominal généralisé, un tympanisme à l'hypochondre droit, un arrêt de matières et de gaz. Plusieurs auteurs ont rapporté des signes similaires [8, 14, 15]. Nous n'avons pas noté des signes de détresse respiratoire contrairement à d'autres auteurs [4, 16, 17]. Dans l’étude de Huang WC et col, portant sur une série de 13 enfants, les signes gastro-intestinaux étaient suivis des signes de détresse respiratoire chez 23,1% de patients [18]. Dans quelques rares cas, ces signes peuvent s'aggraver et conduire à un véritable tableau d'abdomen aigue [5, 15, 19].

En ce qui concerne le diagnostic, l'interposition du colon (signe de chilaiditi) est définie par la présence de l'air en dessous de l'hemicoupole diaphragmatique droite à la radiographie de l'abdomen. La mise au point diagnostic du syndrome de chilaiditi sur bases des images radiologiques requiert la présence des critères suivants: l'hemicoupole diaphragmatique droite doit êtreélevé au-dessus du foie par l'intestin, le colon doit être distendu par l'air illustrant un pseudopneumopéritoine et la marge supérieur du foie doit être abaissée en dessous du niveau de l'hemicoupole diaphragmatique gauche [20].

Les diagnostics différentiels de ce syndrome impliquent un pneumopéritoine [19, 21, 22]. En plus, les changements des positions du patient ne change pas l'image radiologique contrairement à un patient avec de l'air libre. Deux radiographies de l'abdomen de notre nouveau-né tirées en station debout et couchée n'ont montré aucune variation de la position des gaz. D'après Chateil, il peut arriver que l'angle colique droit, hyperaéré, s'interpose entre le diaphragme et le foie. La coupole diaphragmatique droite devient alors visible partiellement ou en totalité. La signification de cet aspect a fait l'objet de controverses multiples. La tendance actuelle est de réfuter sa nature pathologique (syndrome de Chilaiditi) chez les enfants et de considérer cette image comme un aspect normal, apparaissant de façon intermittente à la faveur d'une hyperaération colique [23]. L’échographie a permis de confirmer la présence de gaz dans l'espace hepatodiphragmatique. Cet examen est aussi demandé pour la rechercher d'autres malformations digestives associées [24]. Comme pour la radiographie, à l’échographique, le changement de position du patient n'entraine pas un changement dans la localisation des gaz contrairement à un patient avec pneumopéritoine [19]. Si ces deux examens ne permettent pas de poser le diagnostic, le scanner est recommandé pour établir un diagnostic de certitude.

Le diagnostic différentiel inclue toutes les entités ayant une interposition intestinale comme un pneumopéritoine [19]. Le syndrome de chilaiditi peut être considéré comme une rare cause d'occlusion intestinale. Les pseudoobstructions coliques (ogilvie syndrome) sont aussi observées chez le patient avec syndrome de chilaiditi [9].

Le traitement du syndrome de chilaiditi est souvent conservateur consistant en l'hydratation, la décompression, les laxatifs et l'observation. Des radiographies répétitives lors de la décompression abdominale peuvent montrer la disparition de l'air en dessous du diaphragme. Ainsi, le suivi radiologique de la décompression intestinale peut confirmer à la fois le diagnostic de la maladie et le succès du traitement par la disparition de l'air sous diaphragmatique et par le repositionnement des anses distendues qui retournent à côté du foie. Mais si les douleurs persistent avec développement des signes d'abdomen aigu, l'opération est indiquée [25]. Dans la série de Huang [18], 25%des patients ont subi une correction chirurgicale.

## Conclusion

Le syndrome de Chilaiditi, bien que rare dans l'enfance,mérite d’être évoqué chez un nouveau-né présentant des signes de subocclusion intestinale malgré le nombre important des diagnostiques différentiels. La radiographie de l'abdomen permet de poser le diagnostic. Le traitement chirurgical est envisageable seulement en cas d’échec du traitement conservateur.
